# Cast Microstructure of a Complex Concentrated Noble Alloy Ag_20_Pd_20_Pt_20_Cu_20_Ni_20_

**DOI:** 10.3390/ma15144788

**Published:** 2022-07-08

**Authors:** Lidija Simić, Rebeka Rudolf, Peter Majerič, Ivan Anžel

**Affiliations:** 1 Faculty of Mechanical Engineering, University of Maribor, Smetanova ulica 17, 2000 Maribor, Slovenia; rebeka.rudolf@um.si (R.R.); peter.majeric@um.si (P.M.); ivan.anzel@um.si (I.A.); 2 Zlatarna Celje d.o.o., Kersnikova 19, 3000 Celje, Slovenia

**Keywords:** complex concentrated noble alloy, high—entropy alloy, metastability

## Abstract

A complex concentrated noble alloy (CCNA) of equiatomic composition (Ag_20_Pd_20_Pt_20_Cu_20_Ni_20_–20 at. %) was studied as a potential high—performance material. The equiatomic composition was used so that this alloy could be classified in the subgroup of high—entropy alloys (HEA). The alloy was prepared by induction melting at atmospheric pressure, using high purity elements. The degree of metastability of the cast state was estimated on the basis of changes in the microstructure during annealing at high temperatures in a protective atmosphere of argon. Characterisation of the metallographically prepared samples was performed using a scanning electron microscope (SEM) equipped with an energy dispersive spectrometer (EDS), differential scanning calorimetry (DSC), and X–ray diffraction (XRD). Observation shows that the microstructure of the CCNA is in a very metastable state and multiphase, consisting of a continuous base of dendritic solidification—a matrix with an interdendritic region without other microstructural components and complex spheres. A model of the probable flow of metastable solidification of the studied alloy was proposed, based on the separation of L—melts into L_1_ (rich in Ni) and L_2_ (rich in Ag). The phenomenon of liquid phase separation in the considered CCNA is based on the monotectic reaction in the Ag−Ni system.

## 1. Introduction

The constantly growing demand for high—performance metallic materials presents the driving force for the continuous searching of new, mostly more complex alloys, as well as novel processing strategies [1]. The concept of discovering the complex concentrated alloys (CCA) has made it possible to fill the gap in the strategy of developing the alloys, and also enabled the design of a new class of metallic materials with new and better combinations of properties [2]. Contrary to the commercial alloys, which consist of one principal element and small additions of several alloying elements, the CCAs consist of several principal elements [3]. In fact, the chemical compositions of this group of alloys cover the central regions in phase diagrams. Therefore, metallurgists are nowadays facing the great challenge of discovering an incredibly large number of new alloys that, to date, belong to uninvestigated parts of multicomponent phase diagrams.

**Figure 1:**
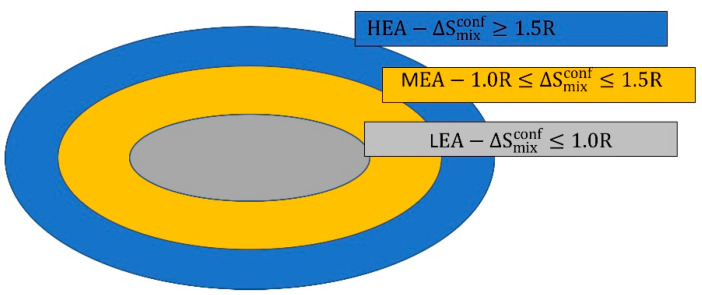
Classification of the alloys according to the configurational entropy of mixing [8].

The group of CCAs consists of several subgroups; one of the most interesting is the subgroup of high—entropy alloys (HEAs), which includes those CCAs with a high configuration entropy of mixing—the microstructure consists of only one single—phase solution (SS). This subgroup of alloys attracts more and more attention due to specific combinations of properties. The first appearance of HEAs occurred in 2004 with the development of a crystalline multicomponent alloy [4]. The characterisation of these alloys caused a special observation because, unlike the Gibbs phase rule, far fewer phases are formed in them. Also, in individual compositions, each element has an equal probability of inhabiting a given network site [4]. HEAs consist of at least five principal elements, where the concentration of each constituent varies across a range from 5 to 35 at. % [5]. The most important criterion for classifying an alloy into the HEA subgroup is the configurational entropy of mixing:

(1) $${\Delta S}_{\mathrm{mix}}^{\mathrm{conf}}=-R\sum_{i=1}^{n}C_{i}\ln\left(C_{i}\right)$$

where R is the gas constant and C is the mole fraction of the i—th element [6]. The maximum value of this parameter is achieved at an equiatomic concentration of constituents and it is calculated as:

(2) $${\Delta S}_{\mathrm{mix}}^{\mathrm{conf}}=R\ln\left(n\right)$$

where n is the total number of constituents [7]. The classification of the alloys according to the configuration entropy of mixing is shown in Figure 1 [8]. The alloys having a configuration entropy greater than or equal to 1.5 R are assigned to belong to the HEA group, the alloys with a configuration entropy between 1.0 R and 1.5 R are classified as media entropy alloys (MEA), while the alloys with a configuration entropy below 1.0 R classified into the group of low entropy alloys (LEA).

From the microstructural point of view, the HEAs are loosely defined as an SS containing five or more principal elements in equal or near equal atomic percentages. The tendency to obtain the single—phase microstructure with a thermodynamically stable SS can be checked for the selected CCA by calculating the parameters that show the ability to form a stable SS. From numerous proposals, the following parameters have been identified in the literature as the most crucial: difference in the atomic size (δ), mixing enthalpy (∆H_mix_), relative entropy effect (Ω), and the valence electron concentration parameter (VEC) [9]. Statistical analyses of experiments for cast HEA led to the criterion for the formation of an SS phase (SS) if δ < 6.2% and −12 kJ/mol < ∆H_mix_ < 5 kJ/mol, while the intermetallic phases (IM) form when δ > 3% and ∆H_mix_ < 0 kJ/mol. HEAs with a mixing enthalpy range that overlaps between the solid solution and the intermetallic phase are grouped into amorphous HEAs [10]. To separate the SS from the IM phase fields, Yang and Zhang proposed the parameter Ω. The SS will be formed at Ω ≥ 1.1 and δ < 3.6%, while the SS + IM will be formed at 3.6% ≤ δ < 6.6% and 1.1 ≤ Ω ≤ 10, whereas at Ω ≥ 10, only the SS has been identified [10]. VEC is mainly used to predict the crystal lattice of a realised phase or phases. For VEC > 8.0, FCC can be formed and for VEC < 6.87, BCC is preferred, while in the cases where 6.87 ≤ VEC < 8.0, a mixture of FCC and BCC is the most possible [9]. On the one hand, these parameters allow researchers to predict the occurrence of a thermodynamically stable SS as the only phase, but, on the other hand, we have to take into account that practically all microstructural characterisations of the potential HEAs are performed in the as—cast state. The most common methods of preparation of these alloys are: vacuum arc melting, induction vacuum melting, mechanical alloying, directional solidification, etc. Newer methods of preparation are the synthesis of high gravity combustion and the production of additives [11]. However, as the castings of the alloys take place mostly uncontrollably, where the solidification path deviates from the equilibrium path, the obtained cast microstructures are metastable with the energy of the local minimum, but not the global one [12]. Accurate determination of thermodynamically stable microstructures and phases requires extremely long—term annealing, as the diffusivity in these alloys is very low. Therefore, the cast microstructures with an SS in the potential HEAs are metastable and the criteria for the formation of an SS based on the experimental results present, in fact, indicators of the ability to achieve a metastable SS.

Previous research in the field of noble HEAs represents relatively new findings in the field of science. Only a couple of scientific papers [13,14,15,16] have been observed in the literature, which mainly deal with a single—phase PdPtRhIrCuNi type of alloy. In these papers, the rules for the formation of the SS phase [16], the development of nanoparticles of this type [13], the assumptions of mechanical properties [15], as well as the electrocatalytic use [17], are presented.

HEAs have very broad applications due to their unique combination of functional and mechanical properties. Potential functional properties are in the field of soft magnets, catalytic materials, superconducting materials, three—dimensional printing, etc. [18].

Based on previous research and calculated parameters for the formation of phases, we casted a new CCNA (Ag_20_Pd_20_Pt_20_Cu_20_Ni_20_), which is being investigated for the first time. The chemical composition of the alloy was selected under the assumption that this alloy possesses high catalytic potential [19,20,21] as well as a high configurational entropy of mixing (∆S_mix_^conf^ = 1.61 R).

According to the comparison of calculated parameters for phase formation (δ = 9.91%; ∆H_mix_ = −3.6 kJ/mol) in the CCNA with the condition for the formation of an SS, it can be assumed that the realised cast microstructure will be multiphase. The basic parameters that enable the prediction of the phase that will be formed in a thermodynamically more stable state were also calculated. Based on previous investigations, in order to predict the phase or phases of the crystal lattice of this alloy, the VEC [22] parameter of 10.32 was calculated, after which it can be assumed that the FCC phase or phases will form. However, in an HEA with noble metals, the enthalpy of mixing of individual pairs plays an important role in the formation of phases [16].

The anomalous casted microstructure similar to those detected in AlxCrCuFeNi_2_ [23] was achieved in our CCNA. Therefore, the ultimate goal of this paper is to present the solidification path with a model prediction. Using different characterisation methods, a model for the solidification of a metastable cast CCNA is proposed.

## 2. Materials and Methods

The CCNA alloy was prepared in an induction furnace under pressure of argon, by melting the pure components (Ag granules—99.99%; Pd sponges—99.99%; Pt sponges—99.99%; Cu granules—99.9%; Ni tiles—99.99%), after which no oxidation was detected. Melting was performed at 1400 °C for 15 min under argon. The liquid melt was cast in a heated iron mould.

A piece of the cast specimen was diffusion annealed in a horizontal tube furnace at 900 °C for 336 h in an argon atmosphere.

The specimens in as—cast and annealed conditions were prepared for metallographic observation by a standard metallographic method (cutting, grinding, polishing, and etching). Cold and warm etching at 70 °C with 5 mL HCl + 10 mL H_2_O + 1 mL HNO_3_ was performed to reveal the microstructure of the CCNA.

The metallographically prepared specimens were examined and analysed by scanning electron microscopy (SEM), equipped by an energy dispersive X—ray spectrometer ((EDS) INCA 350 (Oxford Instruments, Abingdon, UK)), (FEI Sirion 400 NC, FEI Technologies Inc., Hillsboro, OR, USA). Six EDS analyses were performed and only average results are shown. Focussed ion beam (FIB) (Quanta 200 3D, FEI Company, Hillsboro, OR, USA) was used to characterise the matrix/sphere interface and to reveal the microstructure in the core of the sphere using a 30 kV ion current, 5–20 nA milling, and 0.30–0.5 nA polishing.

The thermal behaviour of the cast CCNA was analysed using a differential scanning calorimeter (DSC) STD 650 (TA Instruments (New Castle De, DE, USA)). A piece of the sample was heated and cooled at a rate of 20 °C/min and a second piece of the sample was heated at a rate of 0.5 °C/min while being cooled uncontrollably.

XRD analysis of both cast and annealed samples was performed for the detection of crystallographic phases. X—ray powder diffraction—a Panalyitical X’pert Pro PW 3040/60 goniometer (Malvern Products, Malvern, UK)—was used to measure 2θ between 0° and 110° with a step size of 0.002° and a scan step time of 50 s on each recorded step with Bragg−Brentano optics. The Cu anode with Kα = 0.154 nm was used with a current of 40 mA and a potential of 45 kV. The sample size was an approximately 1 cm^2^ exposed area without previous preparation. 

## 3. Results

### 3.1. Microstructure of Cast CCNA Alloy

**Figure 2:**
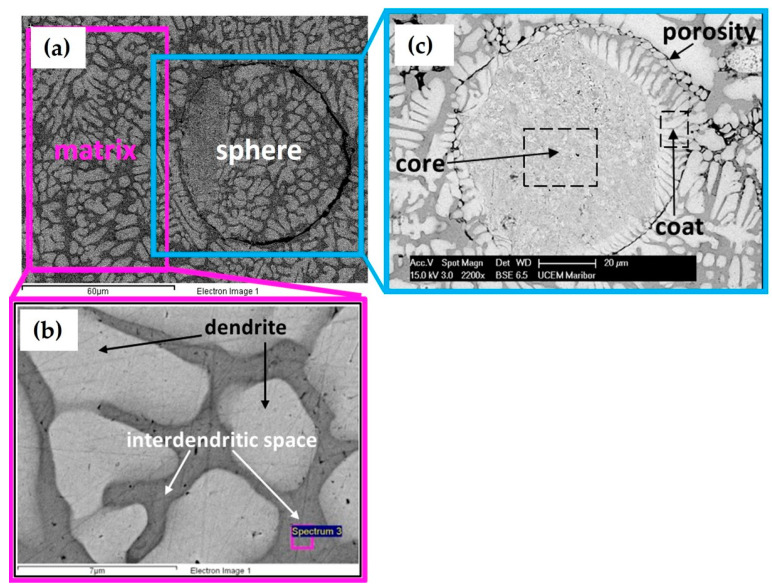
Cast microstructure of Ag_20_Pd_20_Pt_20_Cu_20_Ni_20_ (20 at. %) (**a**) Matrix and sphere (**b**) Sphere with centre and coat (**c**) Matrix with dendrite and interdendritic space.

Figure 2 shows the microstructure of the cast CCNA (Ag_20_Pd_20_Pt_20_Cu_20_Ni_20_) after induction melting under pressure of argon. The CCNA microstructure consists of a matrix and spheres (Figure 2a). The obtained investigation revealed that the matrix is composed of dendrites, with an average size from 2.5 µm to 30 µm and an interdendritic space (Figure 2b). The sphere consists of a core and a coat made of fine dendrites, with an average size from 10 µm to 80 µm, as is visible in Figure 2c. Additionally, a porosity was observed between the coat of the spheres and the matrix.

**Figure 3:**
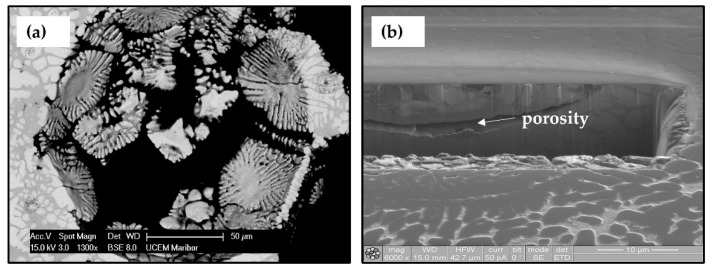
Presentation of the hole and crack (**a**) SEM presentation of the hole formed by the dropout of the sphere and (**b**) FIB presentation of the crack.

After metallographic preparation, spherical holes were observed with the naked eye. Figure 3a shows the growth of matrix dendrites nucleated on the surface of the sphere and the hole was formed by falling out the sphere out of the matrix during metallographic preparation. FIB analysis of this area was performed in order to understand better the loss of spheres from the matrix.

In Figure 3b, which was obtained by ion milling and polishing of the sphere coat, porosity formed in the area of the interdendritic space between the dendrite of the sphere coat and the dendrite of the matrix can be seen clearly.

The resulting different morphology of dendrites in the matrix as well as in the coat of the spheres indicates that differences in composition can have a great influence on the character of the dendrites and growing spheres. The average value obtained from six spectrum EDS analyses of all elements in individual fields are shown in Table 1.

EDS analysis of the cast CCNA shows that different concentrations of all elements have been achieved in the sphere and in the matrix. It was discovered that the coat and matrix have an identical chemical composition. The biggest difference in composition was observed in certain parts of the matrix. The interdendritic space was enriched by Ag (>50 at. %) and Ni was not detected, while dendrites were depleted of Ag (<4 at. %) and rich in Ni (>30 at. %). A clear tendency of the aggregation of elements in the alloy was observed, with one area rich in Ag but depleted of Ni and the other area rich in Ni but depleted of Ag. From the achieved results, the presence of three crystallographic phases can be assumed, of which the first phase is rich in Ni and depleted of silver (dendrite), the second phase is rich in Ag and depleted of Ni (interdendritic space), and the third phase is rich in Ag (the core of the sphere).

### 3.2. Microstructure of the Annealed CCNA Alloy

**Figure 4:**
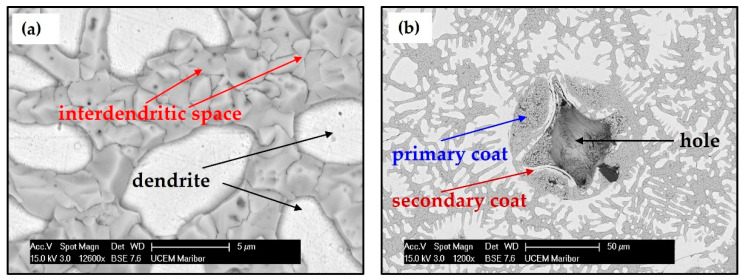
Annealed (900 °C for 336 h) CCNA microstructure (**a**) Dendrites and interdendritic space of the matrix (**b**) A hole with coats.

Determination of the CCNA microstructure approaching the thermodynamically stable state has been performed by long annealing treatments. The achieved microstructure after annealing in an argon atmosphere at 9000 °C for 336 h is shown in Figure 4.

The microstructure consists of a matrix and spheres. The matrix of the annealed sample is built similarly to the matrix of a cast sample, however, a difference was observed in the interdendritic space that contains 100 at. % of Ag, while the dendrites do not have Ag (Figure 4a). However, in the heat—treated sample, the coat of the spheres separated into two parts: the primary and the secondary coat (Figure 4b).

The primary coat has an identical chemical composition to the interdendrite space, as presented in Table 2, while the secondary coat is similar in composition to the dendrites. From the results, it can be assumed that the achieved microstructure of the annealed CCNA is two—phase.

### 3.3. DSC Analysis of the Cast CCNA Alloy

**Figure 5:**
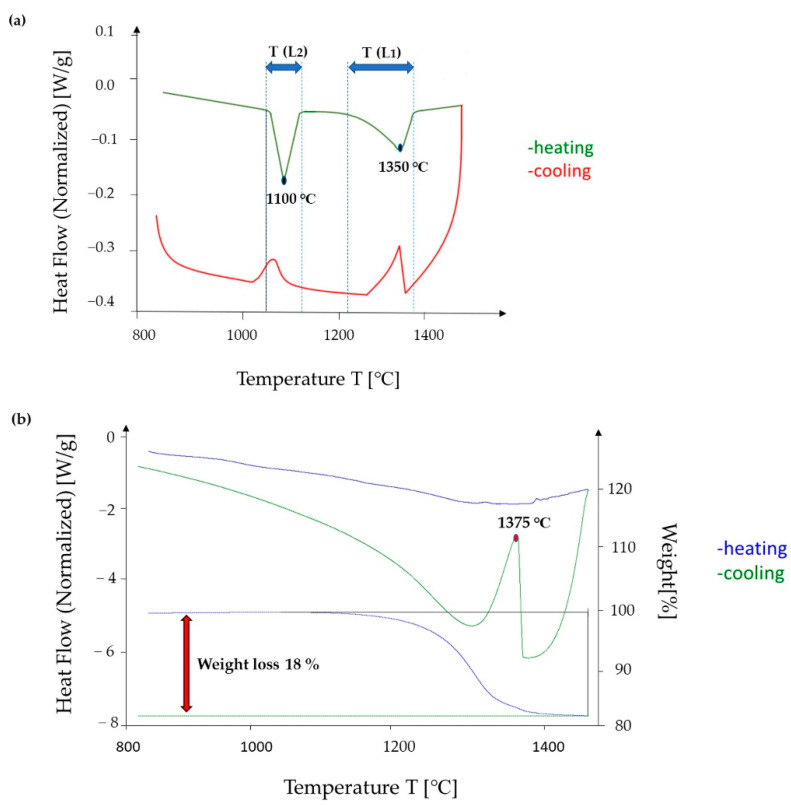
Differential scanning calorimetry curves for casted CCNA at a heating and cooling rate of (**a**) 20 °C min, the green curve indicates heating, while the red indicates cooling (**b**) 0.5 °C min, the blue curve indicates heating, while the green indicates cooling.

The thermal stability and phase evolution in the cast CCNA were analysed by DSC and different heating and cooling rates. Figure 5a shows the DSC thermogram at a heating and cooling rate of 20 °C min in the temperature range from 800 °C to 1600 °C Two sharp peaks (T (L_1_) and T (L_2_)) were observed. The first melting takes place in the temperature range of 1033 °C to 1090 °C T (L_2_), with the sharpest peak at 1010 °C. The second melting takes place in the temperature range of 1160 °C to 1370 °C (T (L_1_)), with the sharpest peak at 1350 °C.

In order to reveal the melting and solidification of the matrix and sphere, a new DSC analysis has been carried out by heating and cooling at very low rate (0.5 °C/min) in the temperature range of 800 °C to 1450 °C. During heating, no broad endo effect was observed and a weight loss of 18% was detected as a result of metal evaporation. However, the cooling curve contained one peak at a temperature of 1375 °C (Figure 5b). In order to determine the element that evaporates at such slow heating, the sample was examined by EDS analysis immediately after DSC analysis.

The obtained results of the EDS analyses show the following chemical composition of Pd_25_Pt_25_Cu_25_Ni_25_ (25 at. %) without Ag (Table 3). Ag evaporated due to slow heating and a high vapour pressure at high temperatures [24]. Based on the fact that the achieved chemical composition (Pd_25_Pt_25_Cu_25_Ni_25_) corresponds to the chemical composition of the matrix dendrites, it follows that the detected solidification temperature corresponds to the solidification temperature of the matrix dendrites.

From the achieved results and by comparing these two thermograms, it can be assumed that the melting temperature T (L_1_) corresponds to the melting temperature of the matrix dendrites and the melting temperature T (L_2_) corresponds to the melting temperature of the sphere.

### 3.4. XRD Analysis of the Cast CCNA Alloy

**Figure 6:**
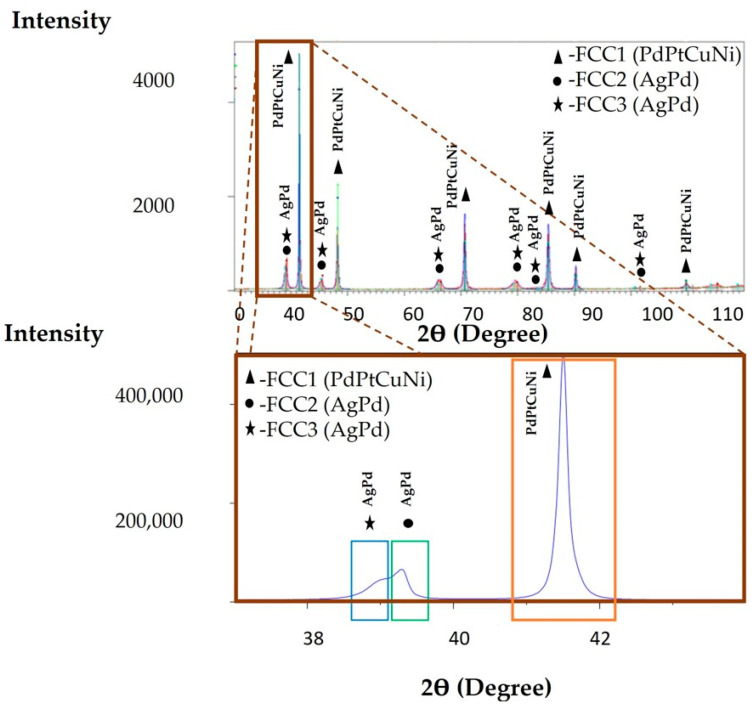
XRD analysis of the cast CCNA sample and XRD analysis in the range 37–44θ.

XRD analysis shows the presence of three crystallographic phases (FCC1, FCC2, and FCC3) in the cast CCNA alloy (Figure 6). In two crystallographic phases (FCC2 and FCC3) whose peaks are very close, the following elements, Ag and Pd, were detected, while the phase marked as FCC1 is rich in Pd, Pt, Cu, and Ni. As no significant difference was observed between the FCC2 and FCC3 phase peaks, the XRD analysis was repeated in the range of 37—44 2θ (Figure 6).

The achieved lattice constant for the FCC1 (rich in Pd, Pt, Cu, and Ni) phase is 3.765 Å; for the FCC2 (rich in Ag and Pd) phase it is 3.996 Å, while for the FCC3 (rich in Ag and Pd) phase it is 3.968 Å. In order to understand which phase (dendrite, interdendrite, or sphere) belongs to a certain XRD peak, a mathematical calculation was applied using Vegard’s law [25].

With the help of Vegards’ law, the individual lattice constants for the following phases were calculated: dendrites, interdendrites, and spheres. The lattice constant is 3.56 Å for the dendrite, 3.95 Å for the interdendritic space, and 3.89 Å for the sphere.

A comparison of the calculated and measured values of the lattice constants is shown in Table 4.

### 3.5. XRD Analysis of the Annealed CCNA Alloy

**Figure 7:**
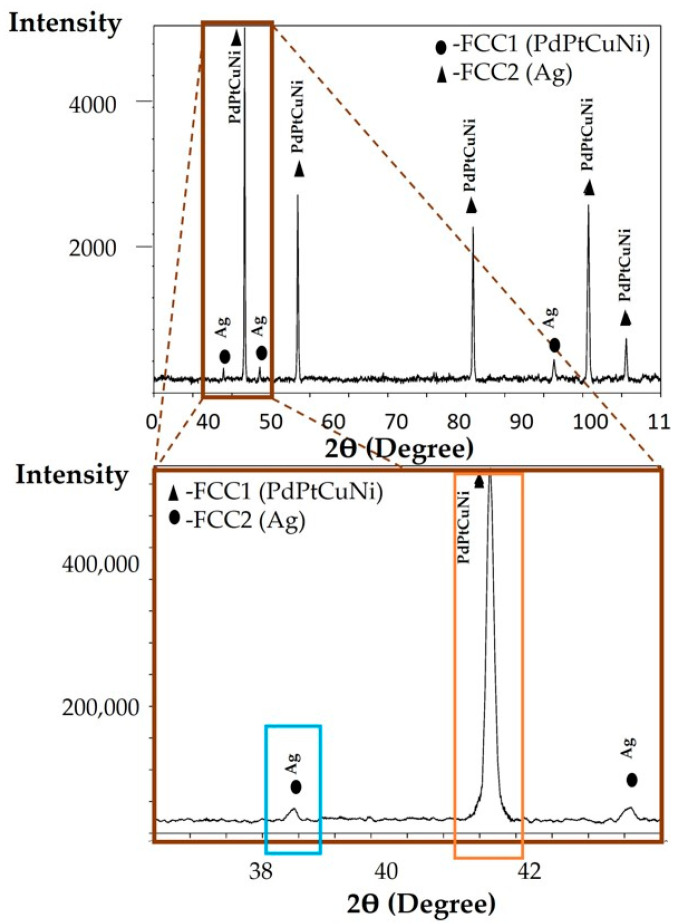
XRD analysis of an annealed CCNA sample and XRD analysis in the range 37–44θ.

XRD analysis, Figure 7, was made on an annealed sample to determine the presence of two phases in the microstructure. The XRD analysis shows the presence of two crystallographic phases (FCC1 and FCC2), with two sharp peaks clearly achieved, which was not the case with the XRD analysis of the cast sample (Figure 6).

The achieved FCC1 phase was rich in Pd, Pt, Cu, and Ni, while containing no Ag at all; the FCC2 phase was composed of almost 100 at. % of Ag. As there are three phases in the microstructure of the cast alloy and two phases in the microstructure of the annealed microstructure, it can be concluded that the system tends to a stable state of two phases by annealing.

## 4. Discussion

The results of our research indicate that the microstructure of the cast CCNA is thermodynamically metastable.

The XRD analysis of the as—cast microstructure confirms three crystallographic phases (FCC1, FCC2, and FCC3). The FCC1 phase is composed of four elements (Pd, Pt, Cu, and Ni), while the other two phases (FCC2 and FCC3) are enriched with Ag and Pd (Figure 6). From the attached comparison in Table 3, as well as the comparison of the lattice constants of the pure metals (Ag = 4079 Å, Pd = 3925 Å, Pt = 3925 Å, Cu = 3628 Å, Ni = 3499 Å), it can be assumed that the FCC1 phase corresponds to the dendrite, which has the lowest Ag (which has the highest lattice constant). The peak of the FCC2 phase encompasses the interdendritic space enriched in Ag with the highest lattice constant, while the peak of the FCC3 phase corresponds to a sphere, also rich in Ag. The XRD results obtained by the annealing sample show the presence of two crystallographic phases, in which one represents the FCC1 or the dendrite, while the other phase, FCC2, represents the core of the sphere and the interdendritic space. The three—phase metastable microstructure has been transformed into a more stable two—phase by simultaneous diffusion of the elements.

The results of the EDS analysis performed on the cast sample, where high concentrations of Ni and Pt in the dendrite and depletion of Ag has been detected by the entire volume of the dendrites, while the interdendritic space is enriched in Ag and Pd and depleted in Ni, indicating that solidification in the CCNA probably starts via a monotectic reaction (L_1_ → α + L_2_).

Because in the alloys of this type, the enthalpy of mixing plays an important role for phase formation, the high concentration of Ag, Pd, and Cu in the interdendritic space region in the cast CCNA alloy can be explained by a positive enthalpy of mixing of +2 kJ/mol, +15 kJ/mol, +2 kJ/mol, +4 kJ/mol between Ag and Cu, Ag and Ni, Pd and Pt, and Cu and Ni [26]. Therefore, from the region of dendrites, Ni and Cu repel Ag and a similar thing takes place in the interdendritic space where higher concentrations of Ag and Pd repel Ni and Pt.

**Figure 8:**
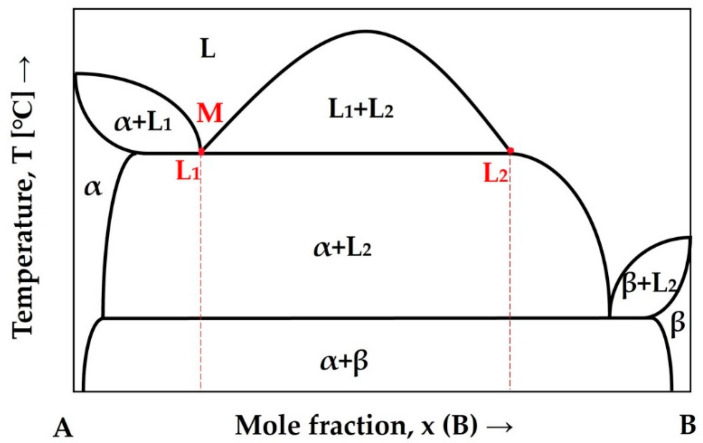
Phase diagram of a monotectic reaction.

Although the CCNA is chemically complex, containing five main elements, the achieved cast microstructure with spheres throughout the volume of the matrix and the two detected melting points in the DSC thermogram indicate that the CCNA can be classified by concentration as a hypermonotectic alloy, as shown for simplicity in Figure 8 for the binary alloying system A—B.

In fact, multicomponent complex alloys of the hypermonotectic type experience liquid phase separation as the temperature of miscibility gap is being reached in the first step of cooling and continues with a monotectic reaction (L_1_ → α + L_2_) in the temperature range where three—phase equilibrium is the source of considerable difference in chemical composition between dendrites (α phase) and the interdendritic space (L_2_).

**Figure 9:**
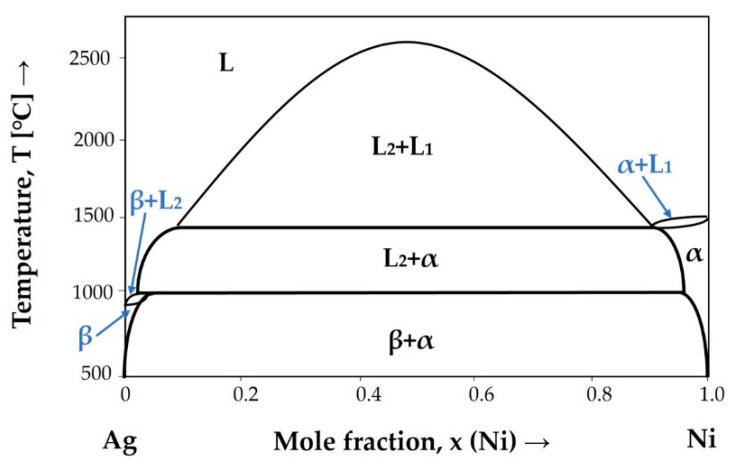
Phase diagram of a two—component Ag−Ni system [27].

We believe that the solidification path of the hypermonotectic CCNA is strongly related to the binary system Ag−Ni (Figure 9) and, therefore, this system served to set up the solidification model for our CCNA.

**Figure 10:**
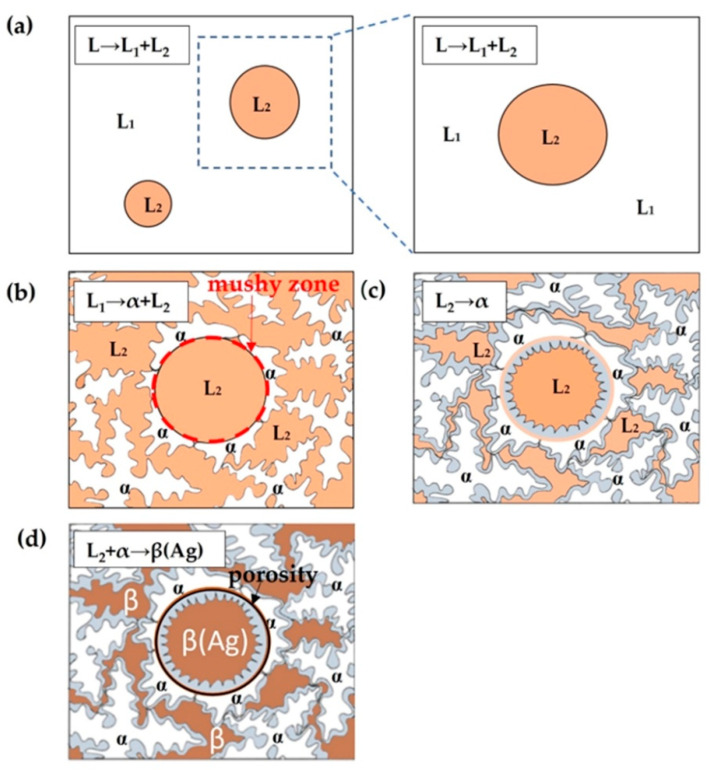
Proposed model of solidification of CCNA (**a**) Liquid phase separation (L → L_1_ + L_2_) (**b**) Monotectic reaction L1 → α + L_2_ (**c**) L_2_ → α solidification of coat (**d**) Peritectic solidification.

The proposed solidification model for the CCNA is shown in Figure 10. The solidification path itself consists of a few steps:

(a) Liquid phase separation L → L_1_ + L_2_;
(b) Monotectic reaction L_1_ → α + L_2_;
(c) Thickening of the dendrites in the matrix and the coat formation in the sphere with solidification of the melt L_2_ (L_2_ → α);
(d) Peritectic solidification of the rest of the melt in the interdendritic space in the matrix, as well as in the core of the sphere.

At the achieved high casting temperatures of the CCNA, the region of liquid immiscibility is reached where liquid phase separation takes place (L → L_1_ + L_2_).

Upon reaching the monotectic temperature, under real conditions, the alloy enters the monotectic space (TMs—TMf) in which the monotectic reaction is stimulated: L_1_ → α + L_2_ solidification of the α—phase with a small concentration of Ag, while the remaining L_2_ melt is rich in Ag.

The α—phase grows in the form of dendrites in the L_1_ melt and form a closed mushy zone around the sphere when the L_2_ melt starts to solidifity into an α—phase (Figure 10b).

**Figure 11:**
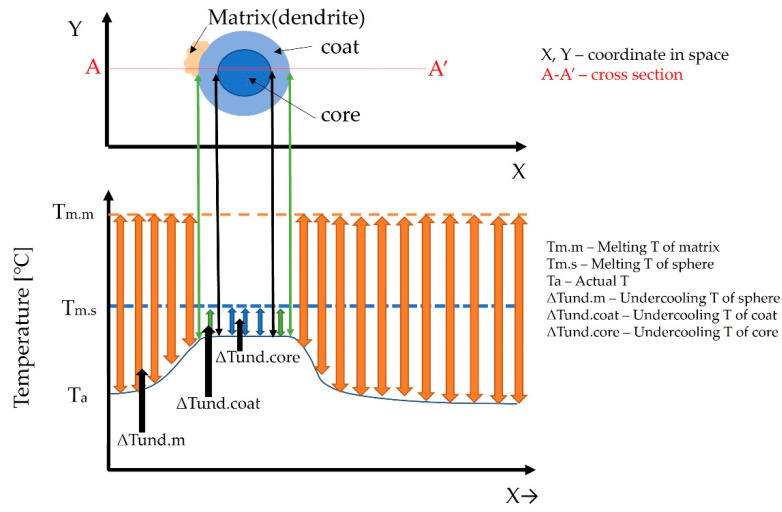
Total undercooling of the sphere with coat and matrix.

With further cooling, the dendrites thicken, while solidification of L_2_ → α takes place on the internal coat of the sphere (Figure 10c). During the solidification of the dendrites in the interior of the sphere, the residual L_2_ melt in the core becomes richer in Ag. At the last solidification stage, the peritectic reaction L_2_ + α → β (Ag) takes place, during which the core of the sphere, as well as the interdendritic space of the matrix have been solidified. The difference in composition between the interdendritic space and the core is due to the closed zone formed by the primary dendrites around the L_2_ melt. The porosity that causes the drop of the sphere from the matrix during metallographic preparations was caused by the lack of melt during shrinkage, due to the complete solidification of the core of the sphere and the matrix (Figure 10d). Based on the previously assumed solidification model, a diagram of undercooling was constructed for the matrix and spheres along A—A‘ (Figure 11).

As can be seen from Figure 11, the melting temperature of the dendrites is higher than the melting temperature of the spheres as a result of which dendrites are the first to solidify. After reaching the appropriate subcooling, the coat of the sphere solidifies, followed by the remaining part of the L_2_ melt.

## 5. Conclusions

The following scientific conclusions can be drawn from the study of the new CCNA (Ag_20_Pd_20_Pt_20_Cu_20_Ni_20_) alloy:

- Cast CCNA is in a highly metastable state with three crystallographic phases present.
- The results of the annealing process show the transition to a thermodynamically stable state with two crystallographic phases.
- Liquid phase separation with a monotectic reaction takes place during CCNA melting, where L melt separates into L_1_ (rich in Ni dendrites) and L_2_ (rich in Ag interdendritic space). As the L_1_ melt achieves greater subcooling, solidification begins with the formation of dendrites around the L_2_ melt, as well as equiaxially.
- As the monotectic reaction of dendrite formation takes place in the space, a further course of solidification is performed by thickening the dendrite and solidifying the coat of the sphere L_2_ → α.
- Solidification of the centre of the sphere takes place via a peritectic reaction, and this represents the last stage.
- The closed mushy zone prevents the L_2_ melt of the interdendritic space and the equalisation of the centre of the sphere, which leads to a small difference in chemical analysis.

## Figures and Tables

**Table 1:** EDS chemical analysis of individual fields (at. %) of the cast sample.

	Components in at. %					
Field		Ag	Pd	Pt	Cu	Ni
Sphere coat	Mean Value	18.43	20.48	18.92	21.28	20.90
	Max. Value	20.41	21.93	20.60	22.97	22.82
	Min. Value	14.83	19.70	17.85	19.93	17.45
	St. Dev.	2.21	0.78	1.09	1.22	2.03
Sphere core	Mean Value	51.83	20.14	2.71	23.24	2.1
	Max. Value	55.29	20.22	2.88	26.12	2.42
	Min. Value	48.37	20.05	2.53	20.35	1.78
	St. Dev.	4.89	0.12	0.24	4.08	0.45
Matrix dendrite	Mean Value	3.45	16.88	29.68	19.28	30.71
	Max. Value	3.95	17.64	31.99	22.50	31.32
	Min. Value	2.82	15.84	26.72	16.48	29.92
	St. Dev.	0.41	0.66	2.30	2.87	0.58
Matrix interdendritic space	Mean Value	54.11	25.05	3.36	17.49	0.00
	Max. Value	56.75	26.83	5.45	20.44	0.00
	Min. Value	52.05	22.52	1.31	15.67	0.00
	St. Dev.	2.11	1.58	1.57	1.96	0.00

**Table 2:** EDS chemical analysis of individual fields (at. %) of the annealed samples.

	Components in at. %					
Field		Ag	Pd	Pt	Cu	Ni
Sphere core	Mean Value	100	0	0	0	0
	Max. Value	100	0	0	0	0
	Min. Value	100	0	0	0	0
	St. Dev.	0	0	0	0	0
Matrix dendrite	Mean Value	0.64	18.58	25.69	27.47	27.62
	Max. Value	3.84	20.88	27.27	29.34	30.61
	Min. Value	0.00	17.53	24.21	20.75	25.07
	St. Dev.	1.57	1.23	1.20	3.31	2.08
Matrix interdendritic space	Mean Value	100	0	0	0	0
	Max. Value	100	0	0	0	0
	Min. Value	100	0	0	0	0
	St. Dev.	0	0	0	0	0

**Table 3:** Chemical composition of a cast CCNA sample after DSC analysis (cooling rate 0.5 °C/min) identified by EDS analyses.

	Components in at. %				
	Ag	Pd	Pt	Cu	Ni
Mean Value	0	25.00	25.00	25.00	25.00
Max Value	0	26.75	25.65	25.85	25.99
Min Value	0	24.99	25.01	25.00	24.95
St. Dev.	0	0.88	0.32	0.43	0.52

**Table 4:** Field lattice parameters obtained by XRD analysis and calculated analysis (Vegard’s law) [13].

Area	XRD Lattice Parameter, a = [Å]	Calculated Lattice Parameter, a = [Å]
Orange area	3765	3.56
Blue area	3996	3.95
Green area	3968	3.89

## Abbreviations

CCNA	complex concentrated noble alloy
CCA	complex concentrated alloy
DSC	differential scanning calorimetry
EDS	energy dispersive spectroscopy
FIB	focussed ion beam
HEA	high—entropy alloy
IM	intermetallic phase
LEA	low entropy alloys
MEA	media entropy alloys
SEM	scanning electron microscope
SS	solid solutions
VEC	valence electron concentration
XRD	X—ray diffraction
